# Antigen rapid tests, nasopharyngeal PCR and saliva PCR to detect SARS-CoV-2: A prospective comparative clinical trial

**DOI:** 10.1371/journal.pone.0282150

**Published:** 2023-02-24

**Authors:** Jean-Marc Schwob, Alix Miauton, Dusan Petrovic, Jean Perdrix, Nicolas Senn, Alexandre Gouveia, Katia Jaton, Onya Opota, Alain Maillard, Gianni Minghelli, Jacques Cornuz, Gilbert Greub, Blaise Genton, Valérie D’Acremont

**Affiliations:** 1 Department of Policlinics, Centre for Primary Care and Public Health (Unisanté), Lausanne, Switzerland; 2 Department of Epidemiology and Health Systems, Centre for Primary Care and Public Health (Unisanté), Lausanne, Switzerland; 3 University of Lausanne, Lausanne, Switzerland; 4 Institute of Microbiology, University Hospital of Lausanne, Lausanne, Switzerland; 5 Vidy-Med, Lausanne, Switzerland; 6 Department of Training, Research and Innovation, Centre for Primary Care and Public Health (Unisanté), Lausanne, Switzerland; 7 Swiss Tropical and Public Health Institute, Basel, Switzerland; Azienda Ospedaliera Universitaria di Perugia, ITALY

## Abstract

**Background:**

Nasopharyngeal antigen Rapid Diagnostic Tests (RDTs), saliva RT-PCR and nasopharyngeal (NP) RT-PCR have shown different performance characteristics to detect patients infected by SARS-CoV-2, according to the viral load (VL)—and thus transmissibility.

**Methods:**

In October 2020, we conducted a prospective trial involving patients presenting at testing centres with symptoms of COVID-19. We compared detection rates and performance of RDT, saliva PCR and nasopharyngeal (NP) PCR, according to VL and symptoms duration.

**Results:**

Out of 949 patients enrolled, 928 patients had all three tests performed. Detection rates were 35.2% (95%CI 32.2–38.4%) by RDT, 39.8% (36.6–43.0%) by saliva PCR, 40.1% (36.9–43.3%) by NP PCR, and 41.5% (38.3–44.7%) by any test. For those with viral loads (VL) ≥10^6^ copies/ml, detection rates were 30.3% (27.3–33.3), 31.4% (28.4–34.5), 31.5% (28.5–34.6), and 31.6% (28.6–34.7%) respectively.

Sensitivity of RDT compared to NP PCR was 87.4% (83.6–90.6%) for all positive patients, 94.5% (91.5–96.7%) for those with VL≥10^5^ and 96.5% (93.6–98.3%) for those with VL≥10^6^. Sensitivity of STANDARD-Q^®^, Panbio™ and COVID-VIRO^®^ Ag tests were 92.9% (86.4–96.9%), 86.1% (78.6–91.7%) and 84.1% (76.9–89.7%), respectively. For those with VL≥10^6^, sensitivity was 96.6% (90.5–99.3%), 97.8% (92.1–99.7%) and 95.3% (89.4–98.5%) respectively. No patient with VL<10^4^ was detected by RDT.

Specificity of RDT was 100% (99.3–100%) compared to any PCR. RDT sensitivity was similar <4 days (87.8%, 83.5–91.3%) and ≥4 days (85.7%, 75.9–92.6%) after symptoms onset (p = 0.6). Sensitivity of saliva and NP PCR were 95.7% (93.1–97.5%) and 96.5% (94.1–98.1%), respectively, compared to the other PCR.

**Conclusions:**

RDT results allow rapid identification of COVID cases with immediate isolation of most contagious individuals. RDT can thus be a game changer both in ambulatory care and community testing aimed at stopping transmission chains, and even more so in resource-constrained settings thanks to its very low price. When PCR is performed, saliva could replace NP swabbing.

**Trial registration:**

ClinicalTrial.gov Identifier: NCT04613310 (03/11/2020).

## Introduction

COVID-19 is responsible for a dramatic health and social situation around the globe. Rapid and accurate detection of SARS-CoV-2 virus in symptomatic individuals is essential for taking immediate measures such as patient isolation and quarantine. Testing is the cornerstone of pandemic management [1]. At present, nasopharyngeal (NP) swabbing followed by reverse transcription RT-qPCR analysis is the reference standard for detection of SARS-CoV-2 infection [2]. This method requires trained staff to perform the swabbing, as well as laboratory personnel and sophisticated equipment to perform PCR analysis. In addition, NP swabbing causes discomfort for the patient and can lead rarely to complications [3]. Also, the turnaround time for getting results is usually 24–48 hours, which forces most tested individuals to wait until they might resume usual activities if tested negative. Another problem of PCR is the persistence of viral detection during several weeks after the end of the COVID-19 episode, when individuals have developed immunity and cannot transmit the virus anymore, as shown in various epidemiological studies [4]. Also, in the vast majority of individuals whose viral load is under 10^6^ copies/ml, the virus is in fact non-culturable, which decreases strongly–but not suppresses–the probability that they can transmit the virus [5–8].

Because of these limitations of PCR, there is definitely a need to investigate alternative testing methods that better reflect transmissibility, to break transmission chains more rapidly and efficiently, release the pressure on the health system and ease the way for patients.

To address the issues of laboratory infrastructure and procedures, as well as turnaround time, several companies have developed point of care antigen rapid diagnostic tests (RDT) to detect SARS-CoV-2. These tests are performed on NP swabs for the time being. Manufacturers report analytical sensitivity above 95% for all of these tests, while independent laboratory based studies report variable performances [9,10]. However, few studies have been performed in the so-called real world, i.e. in clinical settings where these tests will be applied. Results show sensitivities to detect patients with any viral load of 71 to 98%, and specificities between 99% and 100% [11–15].

To address the swabbing issue, there has been several attempts to use saliva for the detection of SARS-CoV-2, as it has been done for different viruses, including coronaviruses responsible for SARS and MERS [16]. For SARS-CoV-2, a systematic review published on studies conducted up to April 2020 documented a sensitivity of 91% (95%CI 80–99%) for saliva and 98% (89–100%) for NP RT-PCR in previously confirmed COVID-19 patients [17]. More recent studies, conducted in hospitalized patients, reported a sensitivity between 80% [18,19] and 100% [20,21].

To simultaneously investigate analytical (PCR and RDTs) and sampling procedures (saliva and NP swab), we conducted a prospective clinical trial in symptomatic patients, in order to compare the detection rate of SARS-CoV-2 and sensitivity of i) RDT on NP swab, ii) PCR on NP swab and iii) PCR on saliva. Secondary objectives were to compare detection rates and sensitivity stratified by VL categories and symptoms duration.

## Methods

### Ethic statement

The study protocol and related documents were approved by the ethical review committee of Canton Vaud (CER-VD 2020–02269). The trial was registered in ClinicalTrial.gov (Identifier: NCT04613310). The authors confirm that all ongoing and related trials for this drug/intervention are registered. All subjects gave a written informed consent in accordance with the Declaration of Helsinki.

### Study design and participants

This was an observational prospective comparative clinical trial. From September 25^th^ to November 4^th^ 2020, patients above 18 years were recruited from three different outpatient sites (Unisanté Bugnon, Unisanté Flon, Vidy-Med) by dedicated staff who were approaching the next patient sitting in the waiting room. Patients were enrolled if they reported having i) at least one major symptom, namely cough, fever, sore throat, anosmia, or ageusia, or ii) a recent close contact with a documented COVID-19 case and presenting with at least one minor symptom (rhinitis, myalgia, headache, fatigue, nausea, vomiting, diarrhoea, abdominal pain, urticaria, vesicles). These criteria corresponded to the regional recommendations for testing (https://coronacheck.ch). Exclusion criteria were unwilling or unable to provide informed consent, or already diagnosed with SARS-CoV-2 in the past, or hospitalized patients, or under anticoagulant therapy.

### Study procedures

Patients collected themselves the saliva for PCR analysis under supervision and according to a video sequence explained step by step by the health professional. Briefly they swabbed their upper and lower gingival space, plus cheeks on both sides, plus under the tongue to end with the hard and soft palate, and this for at least 20 seconds. They finished by drooling twice liquid saliva in the provided 3 ml tube containing universal transport medium, putting the swab in the tube and closing its cap. No coughing or sniffing prior to sample collection was required. Water was avoided 10 minutes prior to collection. Other drinks, food, and nasal sprays were avoided 20 minutes before sample collection. The saliva sampling procedure was chosen after multiple attempts in a pilot study in known negative and positive in- and outpatients.

Then, the health professional collected two nasopharyngeal swabs, one for PCR and one for RDT analyses. Test and control lines were read by the person having collected the swab after 15 to 20 minutes and judged as positive intense, positive weak, or negative. Results were immediately entered into REDCap database. Remaining samples (saliva and the other NP swab) were sent to the molecular diagnostics laboratory for RT-PCR analysis. The patient was considered as positive for SARS-CoV-2 if any of the PCR results or the RDT result on nasopharynx was positive. Due to the nature of the intervention, blinding (to each-other PCR and RDT results) was limited to the molecular diagnosis laboratory. Once identifying information was no longer required for the basic functioning of the trial (i.e. the requirement to identify patient for result return), all eCRFs were strictly anonymised.

### Brand of rapid diagnostic tests evaluated

Three antigen-based RDTs were assessed: 1) STANDARD Q^®^ COVID-19 Ag Test from Biosensor/Roche, 2) Panbio^TM^ COVID-19 Ag Test from Abbott and 3) COVID-VIRO® from AAZ-LMB. All assays are lateral flow tests which detect viral nucleocapsid antigens with color change assessed by naked eye reading. All tests were performed according to manufacturers’ information. RDT brands were rotated (as they could obviously not be performed in parallel on the same patients) after around 30 positive patients until at least 100 positive per test were reached.

### SARS-CoV-2 RT-PCR, cycle thresholds and viral load quantification

SARS-CoV-2 RT-PCR were performed the same day or the next morning using an in-house RT-PCR on the automated molecular diagnostic platform targeting the E gene [22–24], or using the SARS-CoV-2 test of the Cobas 6800 instrument (Roche, Basel, Switzerland). Viral load were obtained by converting cycle thresholds of the RT-PCR instruments, using the formula logVL = - 0.27Ct+13.04, as previously reported [25,26].

### Outcomes

The primary outcomes were the detection rates of SARS-CoV-2 positive patients by PCR on saliva, PCR on NP swab, and RDTs on NP swab, as well as the diagnostic performance (sensitivity and specificity) of i) RDT against NP PCR, ii) saliva PCR against NP PCR, and iii) any test against the other two tests. The secondary outcomes were the viral load of SARS-CoV-2 measured by NP PCR according to RDT results, the viral load by NP PCR and saliva PCR, as well as the diagnostic performance of RDT according to duration and intensity of symptoms.

### Sample size

We based our sample size of 250 positive among 1250 cases tested to have a precision of ±2% on the NP PCR detection rate if the latter was 20% and the power 80%, based on a confidence interval of 95%.

### Statistical analysis

All patients having a result available for the 3 tests were included in the study analysis population. Statistical analyses were conducted using Stata IC 15 (Stata Corp., College Station, Texas, USA) and R statistical software v.4.0.2 (Vienna Austria). Detection rate, sensitivity, and specificity of each test with 95% confidence intervals (CIs) were estimated using standard descriptive methods and subsequently compared using Pearson proportion test for unpaired data, and pairwise proportion tests for paired data. The McNemar test was used to compare PCR and RDT test status (saliva PCR vs NP PCR test status, RDT vs NP PCR test status; paired comparisons). Analyses were stratified by viral load categories. The thresholds chosen for binary stratified analyses by VL were 10^5^ copies/ml (Ct = 30) and 10^6^ copies/ml (Ct = 26). We used Wilcoxon test for comparing nasopharyngeal and salivary viral load (log-transformed) according to RDT test results (positive/negative; unpaired comparisons), RDT band intensity (weak/intense; unpaired comparisons), and saliva volume (low/high; unpaired comparisons). Finally, we used Kruskal-Wallis test for comparing nasopharyngeal viral load (log-transformed) according to RDT brand (STANDARD Q^®^, Panbio^TM^, COVID-VIRO^®^; unpaired comparisons).

## Results

949 patients presumed SARS-CoV-2 providing consent were enrolled. Median age was 31 years (IQR 25–42; range 18–87) with 49% being female. On the day of testing, 96% of participants had at least one major symptom (41% fever, 64% cough, 62% sore throat, 32% anosmia/ageusia) and 4% at least one minor and a close contact with a documented COVID-19 case. Median duration of symptoms at the time of swab collection/testing was 2 days (IQR = 2, range 0–30).

**Fig 1:**
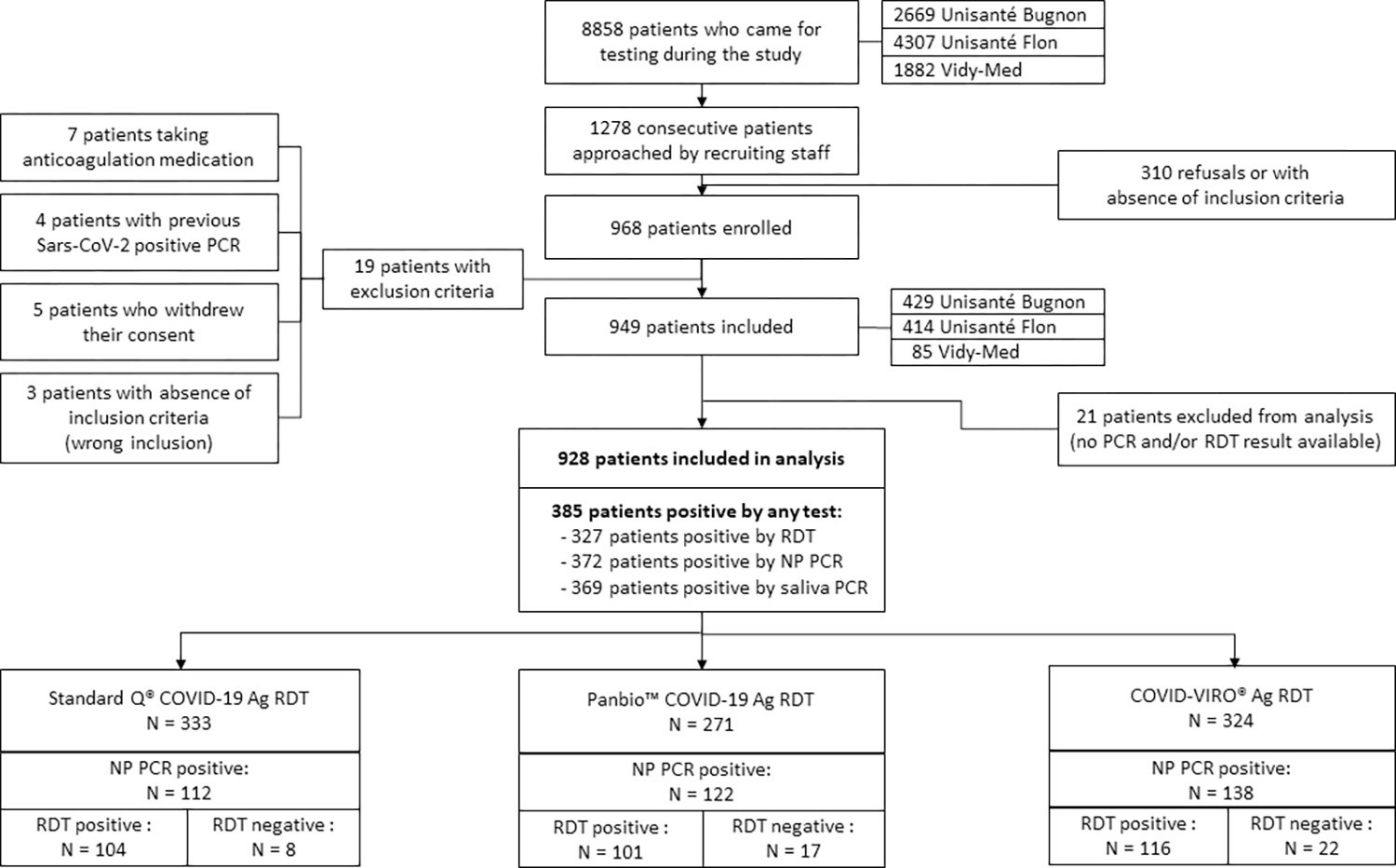
Study patients’ flow.

Among the 928 who had all three tests done, 333 (36%) were tested using STANDARD Q^®^ COVID-19 Ag, 271 (29%) using the Panbio^TM^ COVID-19 Ag, and 324 (35%) using the COVID-VIRO^®^ (Fig 1).

### Detection rates of RDT, NP PCR and saliva PCR

**Fig 2:**
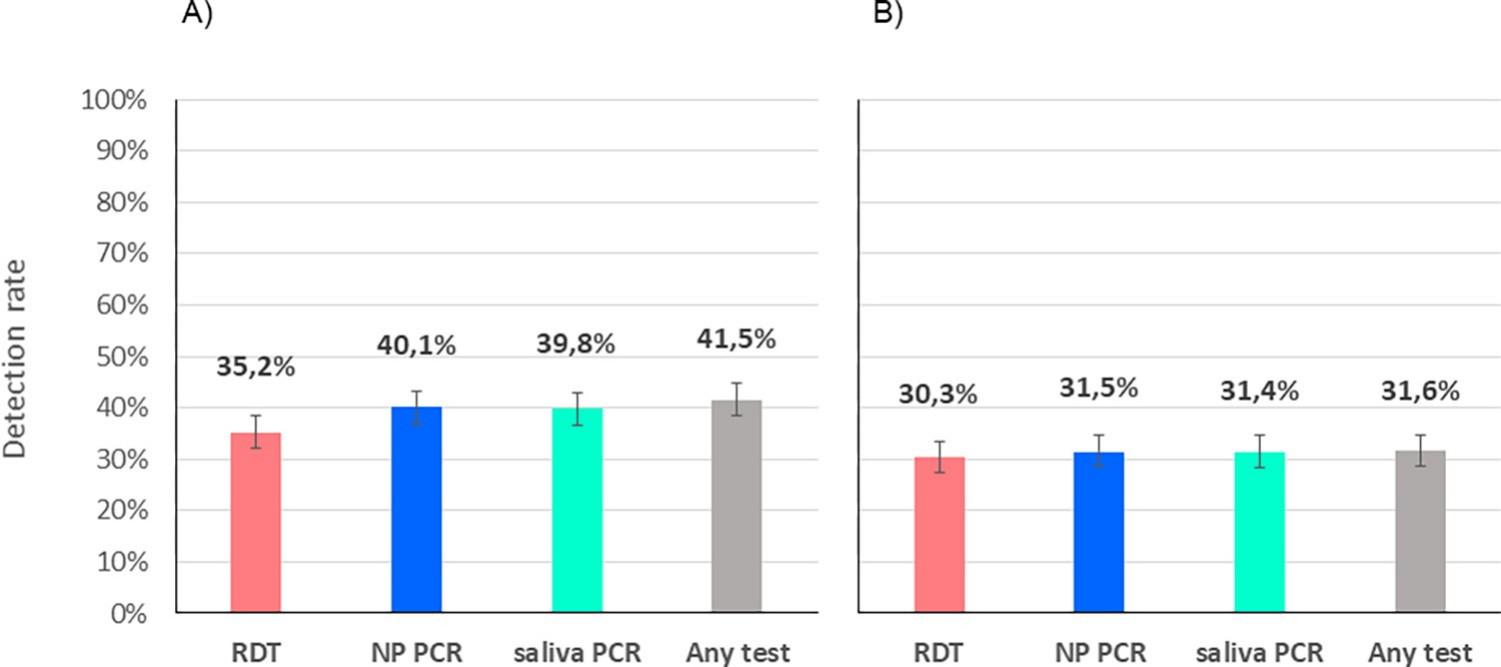
Detection rates of COVID patients by RDT, nasopharyngeal PCR and saliva PCR. A) all positive patients; B) positive patients with viral loads ≥10^6^ copies/ml by any PCR (supposedly significantly contagious).

Of the 928 patients analysed, 327 (35.2%; 95%CI 32.2–38.4%) tested positive by RDT, 369 (39.8%; 36.6–43.0%) by saliva PCR, 372 (40.1%; 36.9–43.3%) by NP PCR, and 385 (41.5%; 38.3–44.7%) by any of the 3 tests (Fig 2A). Detection rates were thus equivalent for both NP and saliva PCR (p = 0.9), with NP PCR detecting 16 additional cases when compared to saliva PCR, and saliva PCR detecting 13 additional cases when compared to NP PCR. The detection rate for RDT was significantly inferior to NP PCR (p = 0.03) and saliva PCR (p = 0.04), with NP and saliva PCR detecting 45 and 42 additional cases compared to RDT, respectively.

When considering the 293 (31.6%) patients with a VL ≥10^6^ copies/ml by either NP or saliva PCR, the differences were lower with a detection rate of 30.3% (27.3–33.3) (281/928) for RDT, 31.4% (28.4–34.5) (291/928) for saliva PCR, 31.5% (28.5–34.6) (292/928) for NP PCR, and 31.6% (28.6–34.7%) by any test (Fig 2B). There were no more significant differences between detection rates of PCRs and RDT (p = 0.6). When considering the 342 (36.9%) patients with a VL ≥10^5^ copies/ml by either NP or saliva PCR, the detection rate was 34.2% (31.1–37.3) for RDT, 36.2% (33.1–39.4) for saliva PCR, and 36.6% (33.5–39.8) for NP PCR (p = 0.4 and 0.3, respectively).

### Diagnostic test performance (sensitivity, specificity) of RDTs, NP PCR and saliva PCR

**Fig 3:**
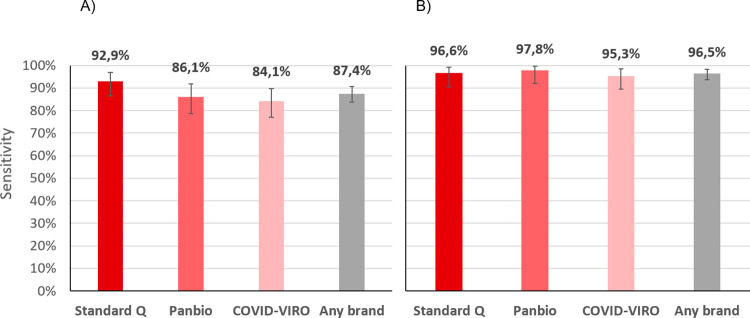
Sensitivity of three brands of antigen RDT compared to nasopharyngeal PCR. A) all positive patients; B) positive patients with viral loads ≥10^6^ copies/ml (supposedly significantly contagious).

The sensitivity of RDT compared to NP PCR was 87.4% (83.6–90.6). When considering those with a VL ≥10^6^ copies/ml by NP PCR, sensitivity was 96.5% (93.6–98.3%) (Fig 3). For those with a VL ≥10^5^ copies/ml, sensitivity was 94.5% (91.5–96.7%).

**Fig 4:**
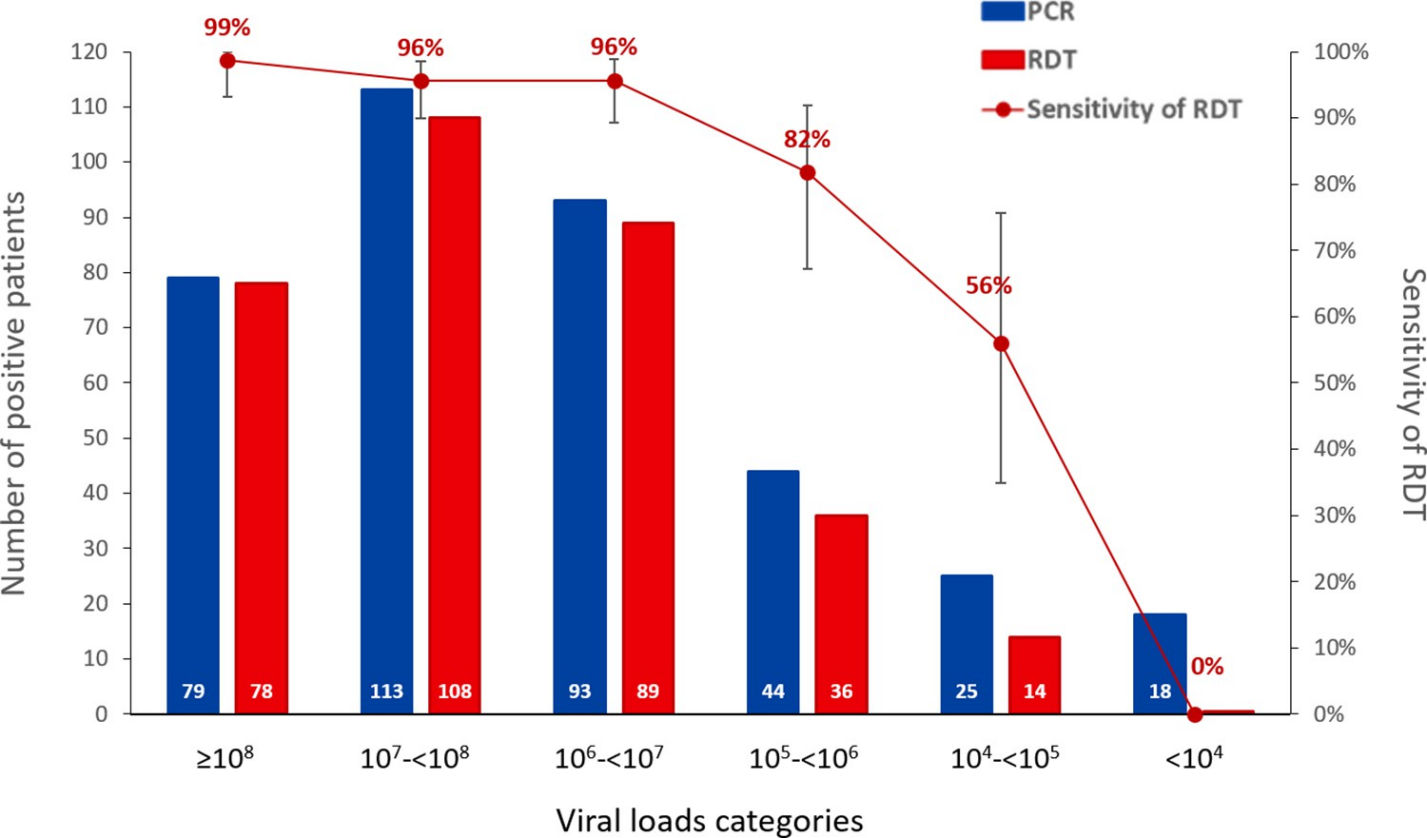
Number of patients positive by RDT (in red) and nasopharyngeal PCR (in blue) and sensitivity of RDT according to viral load categories.

The sensitivity of RDT according to viral load categories remained higher than 95% up to 10^6^ and dropped rapidly for lower loads (Fig 4). No patient with VL<10^4^ was detected by RDT.

**Fig 5:**
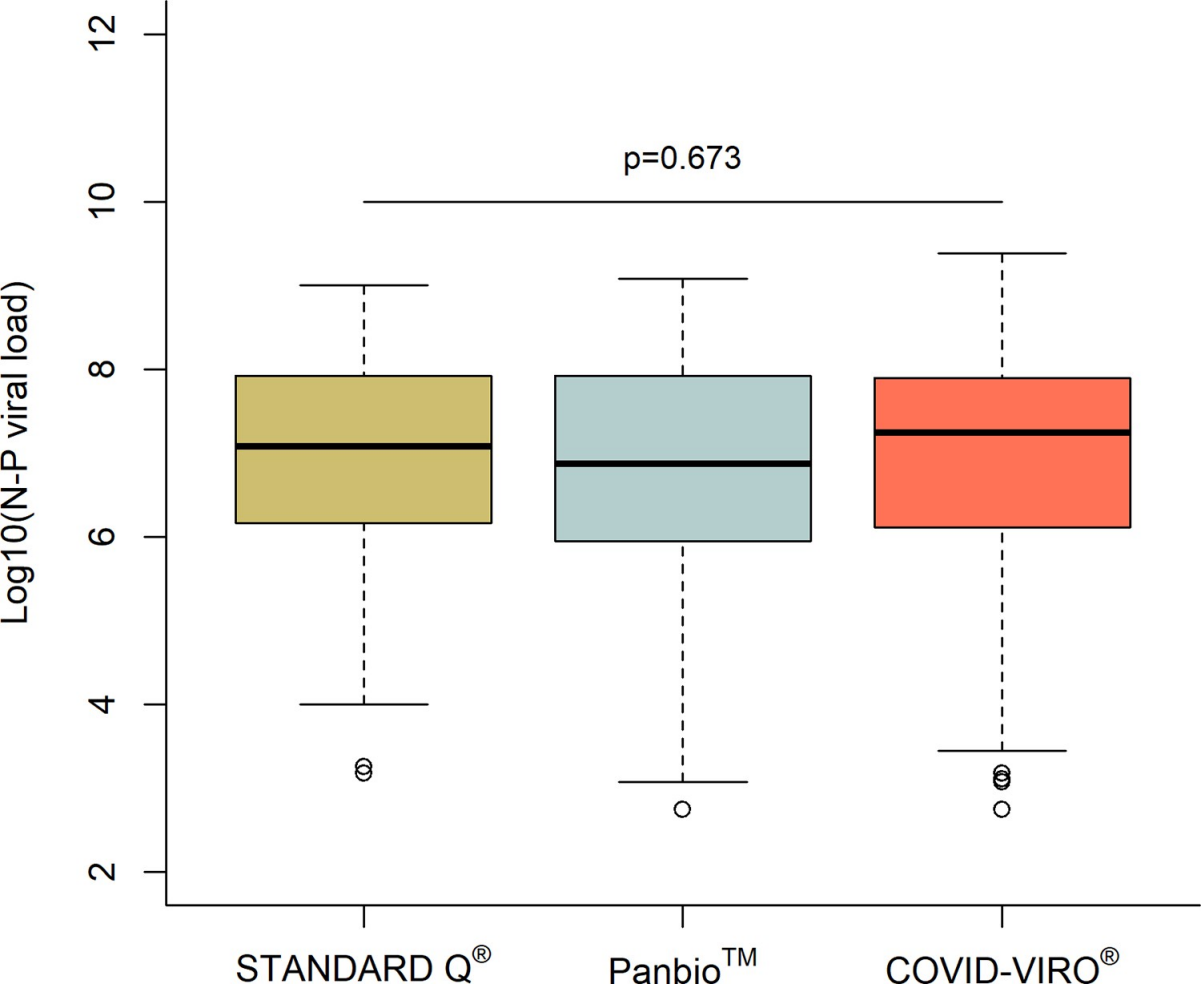
Log viral loads by NP PCR according to the RDT brand used.

Sensitivity of the three RDT brands compared to NP PCR was 92.9% (86.4–96.9%) for STANDARD Q^®^, 86.1% (78.6–91.7%) for Panbio^TM^ and 84.1% (76.9–89.7%) for COVID-VIRO^®^ Ag tests (p = 0.1). When considering those with a VL ≥10^6^ copies/ml by NP PCR, sensitivity was 96.6% (90.5–99.3%), 97.8% (92.1–99.7%) and 95.3% (89.4–98.5%) respectively (Fig 3). Of note, the median log VL of all were similar cases between the 3 brands of RDT (1.2, 0.75 and 1.9 x10^7^ copies/ml respectively, p = 0.67; Fig 5).

The diagnostic performance of NP PCR and saliva PCR were equivalent: sensitivity was 95.7% (93.1–97.5%) for saliva compared to NP PCR and 96.5% (94.1–98.1%) for NP compared to saliva PCR, with an intraclass correlation coefficient (ICC) of 0.935 (95%CI [0.926–0.943]) between these tests.

When using patients with any test positive as reference, sensitivity was 84.9% (81.0–88.3%) for RDT, 95.8% (93.3–97.6%) for saliva PCR, and 96.6% (94.3–98.2%) for NP PCR. The difference was not significant between NP PCR and saliva PCR test status (p = 0.71), but was so between RDT and NP PCR or saliva PCR (p<0.001). Specificity of RDT was 100% (99.3–100%), which means that there was no false positive RDT. In two instances, RDT was positive, and NP PCR was negative, but the saliva PCR was positive with viral loads of 4.0x10^8^ and 1.7x10^3^.

### Comparison of viral loads according to RDT result, and duration and intensity of symptoms

**Fig 6:**
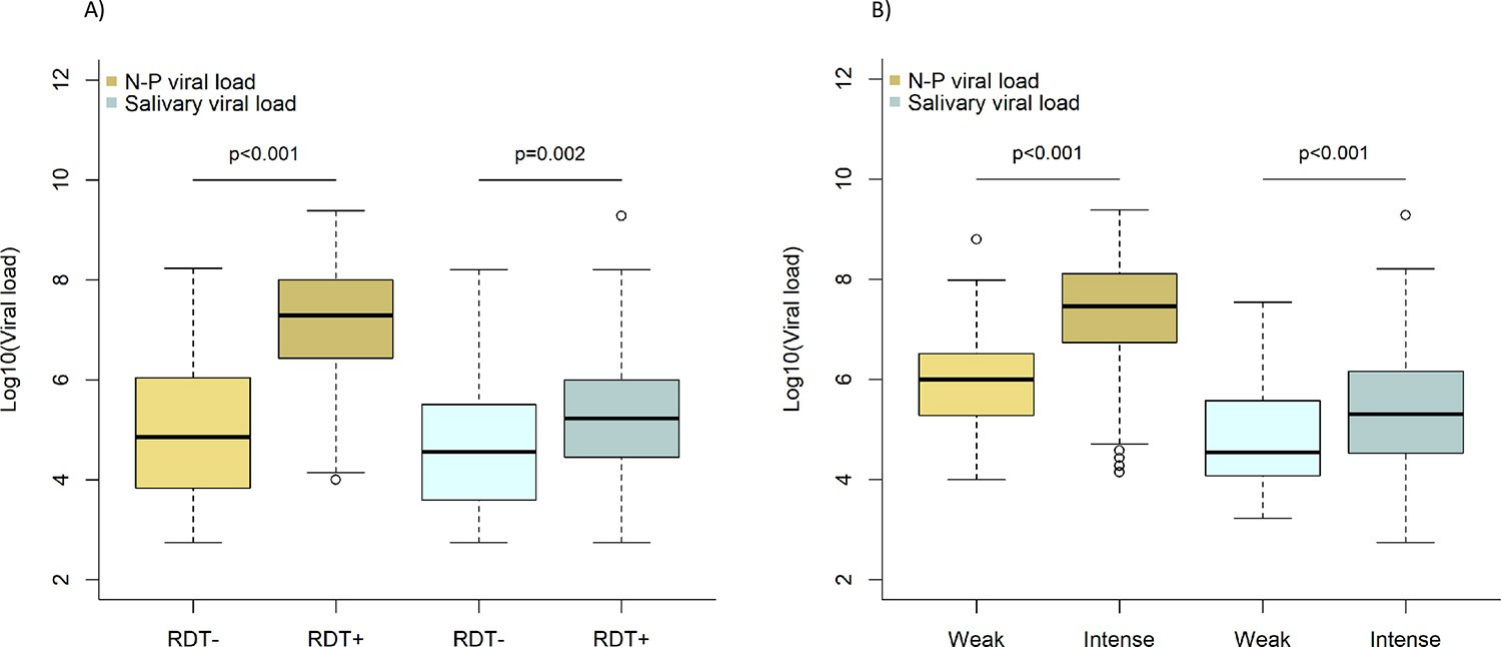
Log viral loads by NP PCR and saliva PCR according to A) RDT result and B) intensity band of positive RDT.

Viral loads (by NP PCR) were significantly higher in patients with RDT positive (median 1.9x10^7^; IQR 2.7x10^6^-1.0x10^8^) than in those with RDT negative (4.2x10^4^; IQR 2.8x10^3^-6.2x10^5^) (p<0.001). Among patients with an RDT positive, those with a colour band of high intensity had also significantly higher viral loads by NP PCR (median 2.8x10^7^; IQR 5.8x10^6^-1.3x10^8^) than in those with a colour band of low intensity (median 7.1x10^5^; IQR 1.5x10^5^-3.2x10^6^) (p<0.001) (Fig 6).

**Fig 7:**
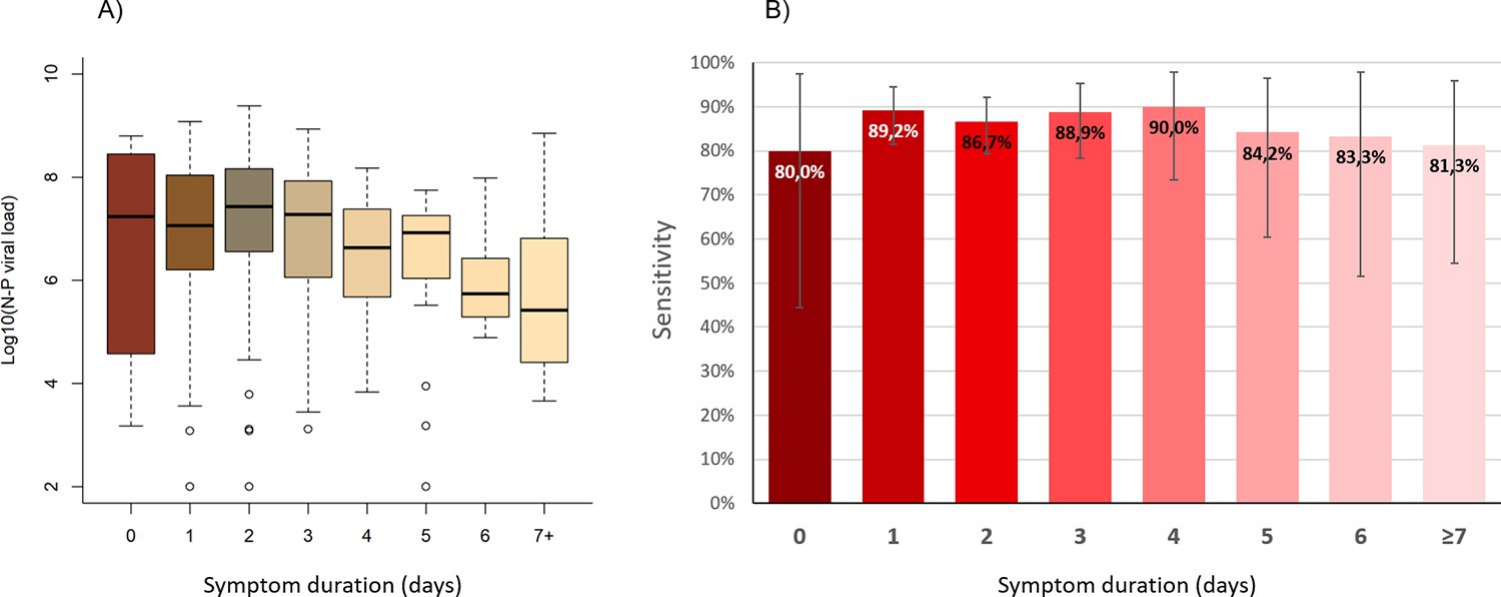
Log viral loads according to symptoms duration by nasopharyngeal PCR (A) and sensitivity of antigen RDT (B).

The sensitivity of RDT according to symptoms duration varied between 80% and 90%. It was lowest the day of symptoms onset (80%, 95%CI 44.4–97.5%) and highest on day 4 (90.0%, 73.5–97.9%) (Fig 7). There was no significant difference in the sensitivity of RDT, neither between the first 4 days of symptoms (87.8%, 83.5–91.3%) and thereafter (85.7%, 75.9–92.6%) (p = 0.6), nor the first 7 days (87.7%) and thereafter (81.3%) (p = 0.5).

Viral loads (by NP PCR) were significantly higher (median 1.3x10^7^; IQR 1.3x10^6^-8.4x10^7^) in patients with major symptoms than in those with minor symptoms only (median 2.9x10^6^; IQR 8.0x10^2^-6.6x10^7^) (p = 0.007), but the number of positive patients with minor symptoms was very small (8).

### Viral loads according to PCR type of sampling

**Fig 8:**
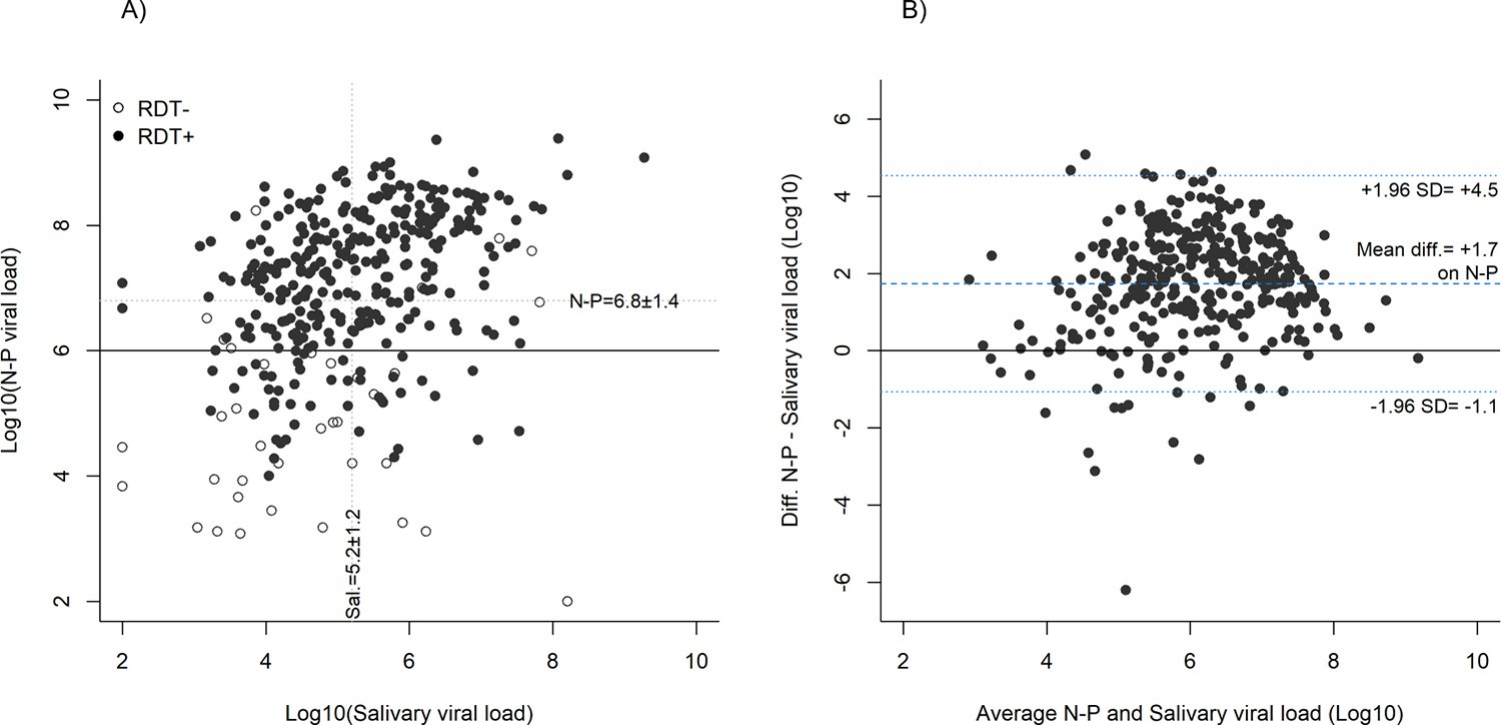
Comparison between log viral loads by nasopharyngeal PCR and saliva PCR. A) Log viral loads in RDT positive (black dots) and negative (white dots) patients. Dotted lines: Mean log viral loads; Black line: Considered threshold for presence of cultivable virus (nasopharyngeal PCR); B) Bland-Altman analysis showing the difference between nasopharyngeal and saliva log viral loads; SD = Standard deviation.

VLs of patients with positive saliva PCR (median 1.3x10^5^; IQR 1.9x10^4^-8.8x10^5^; range 2.0–9.3) were significantly lower than those with positive NP PCR (median 1.3x10^7^; IQR 1.3x10^6^-8.4x10^7^; range 2.0–9.4) (p<0.001). Among 285 patients with VL≥10^6^ by NP PCR, 10 (3.5%) were negative by RDT. Among 87 patients with VL<10^6^ by NP PCR, 50 (57.5%) were positive by RDT (Fig 8A). The difference between NP and saliva VLs is illustrated in Fig 8B.

**Fig 9:**
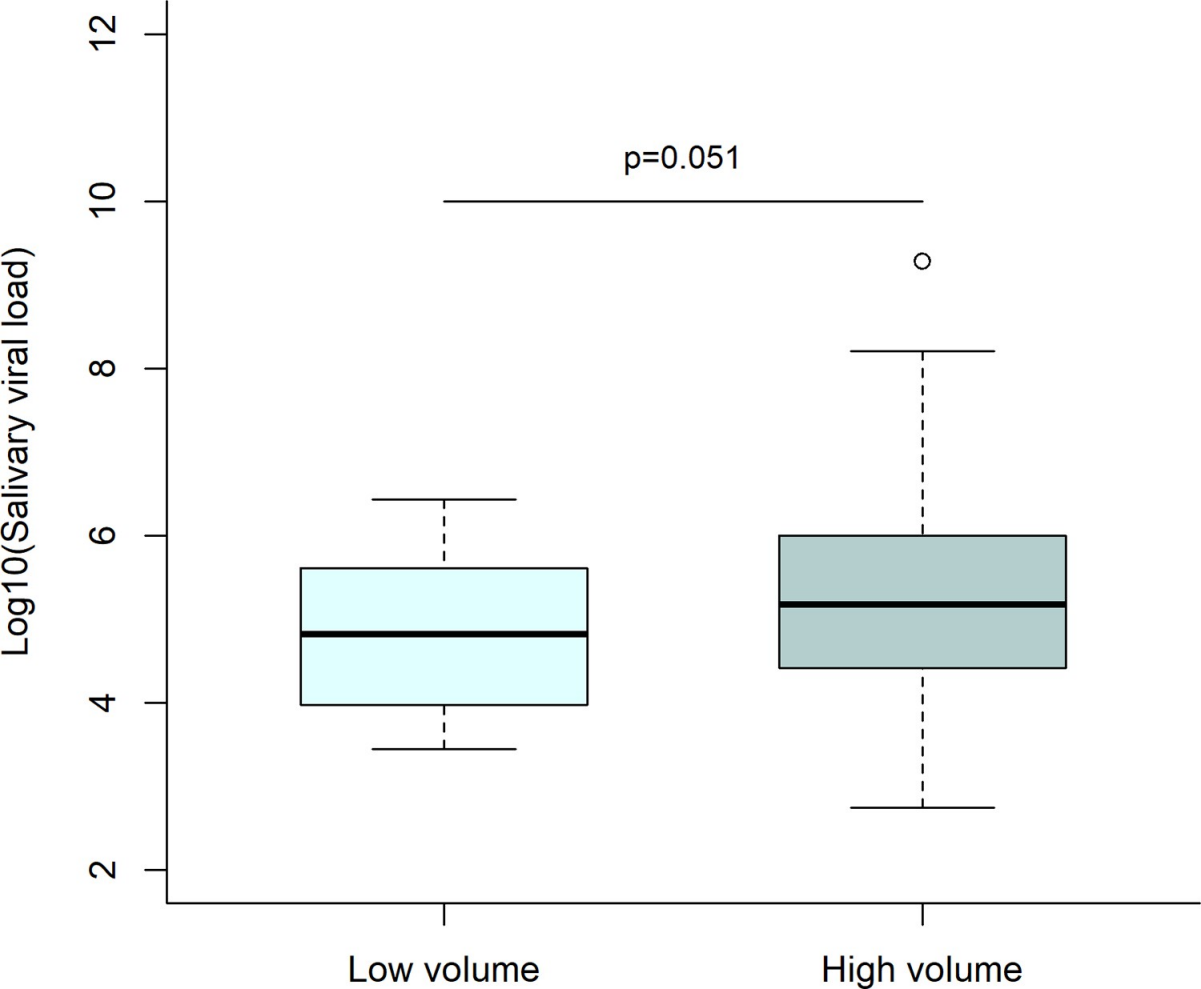
Log viral loads by saliva PCR saliva volume: Low volume corresponds to a gingivo-buccal swab only; high volume corresponds to a gingivo-buccal swab with <0.5 ml of saliva in addition.

Viral loads (by saliva PCR) were not significantly higher in patients with high volume of saliva (median 1.3x10^5^; IQR 2.2x10^4^-9.7x10^5^) than those with low volume (median 9.4x10^4^; IQR 9.6x10^3^-5.1x10^5^) (p = 0.051) (Fig 9).

The two PCR were equivalent also in patients with ageusia or sore throat and no other symptom outside anosmia (p = 0.7).

## Discussion

The results of the present study show that the detection rate of positive COVID-19 cases by RDT was high, especially for those with a VL of ≥10^6^ copies/ml. The sensitivity of RDT compared to NP PCR was above 85%, and above 95% for patients with a high VL, and was similar between the three RDT brands, with an advantage for STANDARD Q^®^. The sensitivity varied slightly according to symptoms duration but remained above 80%, even after 7 days despite a progressive drop in VLs. Actually, a lower sensitivity after the acute phase of disease might be an advantage to prevent unnecessary isolation of patients who are, for most of them, no more contagious, despite a positive PCR result (known to sometimes persist for a long time) [27,28]. Our results are quite strong since they are based on a high number of positive patients and two PCR performed in parallel on each patient. We reached 385 positive cases, with >100 by RDT brand, thus fulfilling the FIND/WHO requirements for validating rapid diagnostic tests.

The detection rate of SARS-CoV-2 by PCR performed on a saliva sample was equivalent to that of RT-PCR performed on NP swabs. The sensitivity of PCR of one type of sampling compared to the other were similar and above 95%. The two positive saliva PCR but negative NP PCR patients who were still detected by RDT illustrates that some but rare false negative results of tests based on NP swabs are likely due to sampling procedure.

### RDT versus PCR

The sensitivity of RDT of more than 95% in patients with VL≥10^6^ copies/ml means that these rapid tests are likely to identify reliably individuals that are contagious, which would, if largely deployed, reduce transmission more substantially than what would be expected from its imperfect overall sensitivity. Furthermore, the short turn-around time to return the result to the patient allows more rapid isolation of cases and efficient contact tracing, which should also contribute to more efficient pandemic control.

There was a slight variability in performance between the three different RDTs with STANDARD Q® having a higher sensitivity (93%) than those of Panbio^TM^ (86%) and COVID-VIRO® (84%), but all met the threshold of 80% sensitivity and 97% specificity of the WHO recommendations for use [29]. The sensitivity of all tests in the present study was in the range of those reported in the other manufacturer-independent clinical validation studies [11–15]. Our high number of patients investigated with well-defined conditions and standardized procedures make results of the present work rather robust. Obviously, because the sensitivity of rapid tests is highly correlated to the viral load, the overall sensitivity found in each study depends on the distribution of viral loads in the included participants. Overall sensitivities can thus not be directly compared between studies and results should always be provided by viral load ranges (Fig 4).

In our study, the specificity of all three tests was 100%, which is impressive considering the potential for inter-observer variation in RDT test line reading. This observation implies that the assessed RDTs brands are easy to read, and that faint lines can still be easily detected. This excellent specificity, which was also shown in the other studies on high quality RDT, allows to state that there is no need to confirm a positive RDT test result by an additional PCR test.

One of the strengths of our study also lies in the fact that the study population represented that of routine COVID-19 diagnostic centres, namely symptomatic outpatients with fever or cough or anosmia/ageusia or symptomatic close contacts. The study was performed at the end-user level in real-life conditions, which is in agreement with WHO recommendations for evaluating the performance of new diagnostic tests [30]. Real-life evaluation also allows to take into account context-specific factors that could influence clinical accuracy, such as common comorbidities.

### Saliva PCR versus NP PCR

Having a detection rate of saliva PCR equivalent to that of NP PCR is in line with a previous study done on 70 patients COVID-19 positive that showed excellent concordance between the two sampling methods [21]. Other studies showed much lower performance with around 80% sensitivity for saliva PCR when compared to NP PCR. These divergent results are likely to be due to the sampling procedure and inhibitors that can interfere with amplification.

The median SARS-CoV-2 VL in saliva was approximately two log lower than that in the NP swab. With such a difference, an overall lower sensitivity of saliva PCR when compared to NP PCR would have been expected. This was not the case in the present evaluation because it is essentially the peak and not the extremes of the VL distribution curve that is shifted towards a lower value for saliva.

In terms of procedures, patients were able to easily perform the saliva sampling on themselves after getting a precise explanation by the health professional. Some were not able though to drool saliva in the tube, but this did not affect the sensitivity, the VL being not significantly lower in this group.

The FDA has granted emergency use authorization to various saliva-based assays for the SARS-CoV-2. Our pragmatic approach, using the same transport medium tube as for NP swabs, can be applied in any testing facility. The similar sensitivity and specificity achieved by sampling the saliva instead of the nasopharynx validates the sampling method and procedure, at least in this outpatient population with relatively high viral load. The results of the present study, together with that of Wyllie *et al* [21] and others [31,32], provide evidence that RT-PCR on saliva can be used for SARS-CoV-2 detection to ease testing and improve comfort and safety.

### Clinical significance

If RDT would be used in settings with a lower SARS-CoV-2 prevalence, such as 10%, a negative test would have a negative predictive value (NPV) of 98.6%. Such an NPV is acceptable if the patients do not belong to high-risk populations (severe cases, hospitalized patients). If the prevalence would be only 1%, the NPV would be 99.9%. Considering a specificity of 100%, other NPV, whenever for RDT or PCR, can be simply calculated using the following formula: (1-P)/1-SP (P = prevalence; S = sensitivity).

Regarding the positive predictive value (PPV), even taking the lowest specificity confidence interval (99.3%), it would be 93% at 10% prevalence, which is high enough. At a lower prevalence, the PPV would drop, and the solution would be to restrict testing to patients with a high enough pre-test probability rather than confirming each positive case by PCR.

### Limitations

The present study was conducted in a well-defined outpatient population with usual testing criteria for COVID-19 and presenting within 7 days after symptom onset for most of them. Our results might not apply in a setting where patients would have lower viral loads and/or attend after one week of symptoms, keeping in mind that these outpatients would be much less likely to transmit [33]. We did not include children in the study; however, viral loads seem not to differ between children and adults [34], which suggests that RDT could perform similarly in younger age groups. We cannot infer the accurate diagnostic performance of saliva PCR and RDTs in an asymptomatic population that was not investigated here. However, the ability of RDTs to detect a person with a sufficient viral load to transmit the virus remains the same, whenever the person is symptomatic or not. Therefore, the sufficient sensitivity (82%) in patients with viral loads between 10^5^ and 10^6^ suggests that RDTs can be safely used for screening schools, university students or contacts of SARS-CoV-2 positive patients [35].

## Conclusion

The good performance of the best available RDTs allows point of care management of patients at primary care level, as well as community testing aimed at stopping transmission chains. RDT results allow immediate isolation of the vast majority of contagious individuals, without confining unnecessarily those who are not. The almost perfect concordance between saliva PCR and NP PCR results, the ease of administration and the safety of the procedure could trigger change in sampling method using saliva as reference standard, at least in outpatients who have higher viral loads than inpatients. RDT complies with the ASSURED (Affordable, Sensitive, Specific, User-friendly, Rapid and robust, Equipment-free and Deliverable to end-users) criteria, which makes them very useful in primary care practices, and, thanks to its very low price, even more so in resource-constrained settings [36]. The variability in performance of different RDTs highlights the need to continue the efforts for having manufacturer-independent validation in the population that will be the target group for use before implementation of a new brand at large-scale.

## Supporting information

S1 File Patient’s database. (PDF)

S2 File Study’s protocol. (PDF)

S3 File Strobe checklist. (PDF)

S4 File TREND checklist. (PDF)

S5 File (XLSX)
