# Supplementary material for: Antigen rapid tests, nasopharyngeal PCR and saliva PCR to detect SARS-CoV-2: A prospective comparative clinical trial
Source: PLoS One. 2023 Feb 24;18(2):e0282150. doi: 10.1371/journal.pone.0282150 (PMC9955963; doi:10.1371/journal.pone.0282150)
Supplement: S2 File — (PDF) [file pone.0282150.s002.pdf]

unisanté
Centre universitaire de médecine générale et santé publique · Lausanne

Clinical Study Protocol

PCR and Rapid Diagnostic Test on saliva and nasopharyngeal swabs for the detection of SARS-CoV-2: a comparative clinical trial (RaDiCo)

Study Type:	Clinical trial
Study Categorisation:	OClin category A & C
Study Registration:	Clinicaltrials.gov<br>Swiss National Clinical trial Portal (SNCTP via BASEC)<br>BASEC number: ID 2020-02269
Study Identifier:	RaDiCo
Sponsor,	**Sponsor:** Unisanté
Principal investigator:	Prof Valérie D'Acremont<br>Unisanté: Département Formation, recherche et innovation<br>& Policlinique de médecine tropicale, voyages et vaccinations<br>M :+41 79 556 25 51
Investigational Product:	None
Protocol Version and Date:	Version 5.0, Oct. 8th 2020

CONFIDENTIAL.

The information contained in this document is confidential and the property of the sponsor. The information may not - in full or in part - be transmitted, reproduced, published, or disclosed to others than the applicable Competent Ethics Committee(s) and Regulatory Authority(ies) without prior written authorisation from the sponsor except to the extent necessary to obtain informed consent from those who will participate in the study.

unisanté
Centre universitaire de médecine générale et santé publique · Lausanne

Signature Page(s)

Study Title : PCR and Rapid Diagnostic Test on saliva and nasopharyngeal swabs for the detection of SARS-CoV-2: a comparative clinical trial (RaDiCo)

The Sponsor have approved protocol V5.0 dated 8 October 2020 and confirm hereby to conduct the study according to the protocol, current version of the World Medical Association Declaration of Helsinki, ICH-GCP guidelines and the local legally applicable requirements.

Prof. Jacques Cornuz

Lausanne, 8.10.2020

Place, Date

Signature

Principal investigator:
I have read and understood this trial protocol and agree to conduct the trial as set out in this study protocol, the current version of the World Association Declaration of Helsinki, ICH-GCP and the local legally applicable requirement.

Site : Unisanté, Lausanne

Principal Investigator : Prof. Valérie D'Acremont

Lausanne, le 8.10.2020

Place, Date

Signature

STUDY SYNOPSIS

**Sponsor**	Unisanté, Lausanne Switzerland
**Study Title:**	PCR and Rapid Diagnostic Test and on saliva and nasopharyngeal swabs for the detection of SARS-CoV-2: a comparative clinical trial (RaDiCo)
**Short Title / Study ID:**	Tests on saliva for the detection of SARS-CoV-2 (RaDiCo)
**Protocol Version and Date:**	Version 5.0, 08.10.2020
**Trial registration:**	This trial is registered in the ClinicalTrials.gov registry and in the Swiss National Clinical trial Portal (SNCTP via BASEC)<br>FOPH Registration number:
**Study category and Rationale**	**OClin category A**<br>- The interventions assessed are the PCR on saliva and the RDT on nasopharyngeal swab against the reference test, which is the PCR on nasopharyngeal swab. RDTs on nasopharyngeal swabs are CE approved and used according to the manufacturer's instructions.<br>**OClin category C**<br>- The intervention assessed is the RDT on saliva against the reference test, which is the PCR on nasopharyngeal swab. RDT is CE approved but its use diverges from the manufacturer's instruction.
**Clinical Phase:**	Diagnostic assessment<br>- Compare the detection rates of PCR on saliva, RDT on nasopharyngeal swab, RDT on saliva against PCR on nasopharyngeal swabs

**Background and Rationale:**	The epidemiological situation of COVID-19 shows a steady increase of patients that fulfil diagnostic testing criteria in Switzerland, and canton of Vaud (VD) in particular. It is imperative to adapt the testing strategy to accommodate a massive influx of patients (~ 2-300/d at Unisanté, >1000/d at VD). Recent literature shows that the sensitivity of the PCR tests for the detection of SARS-CoV-2 using saliva samples is close to that using nasopharyngeal swabs [see summary table from Schwob JM (unpublished) in Appendice 1]. This type of sampling represents a practical advantage since it can be performed by the patient herself/himself and would thus allow to speed up the collection process. It is also less painful and could prevent the rare lesions to the nasal mucosa that can occur when using nasopharyngeal swabs.<br><br>Even if the published papers are rather convincing (Wyllie et al 2020, Kojima et al 2020, Williams et al 2020, Pasomsub et al 2020, Vogels et al 2020) for the PCR on saliva, they are mostly based on laboratory validation. It seems now necessary to move from the laboratory to clinical validation to confirm that the detection rate of PCR tests is not inferior when samples are collected from saliva rather than nasopharyngeal swabs in field conditions. Such a formal clinical validation would allow accurate information to convince the national COVID task force, the health regulation authorities (Swissmedic) as well as the other sectors in Switzerland that the saliva sample is appropriate for the detection of SARS-CoV-2.<br><br>In addition, Rapid Diagnostic Tests for the detection of SARS-CoV-2 antigens have been developed using nasophayngeal swabs and have shown very high sensitivity against PCR, ranging from 93% to 98% when based on laboratory validation. [see summary table from Schwob JM (unpublished) in Appendice 2 and Kruger et al https://www.medrxiv.org/content/10.1101/2020.10.01.20203836v1]. This method offers the considerable advantage to inform the patient of the test result on site, and allow the provision of appropriate recommendations on the spot of testing. The studies performed so far have been conducted using nasopharyngeal samples only. There are no data with saliva yet. It is expected that the RDT would also work on the saliva. Even if slightly less sensitive due to the fact that it detects antigens and not multiplied RNA as PCR does, RDT on saliva could better serve the public health goal to test widely and quickly and have ultimately more COVID cases detected and isolated, and hence reduced transmission.<br><br>To investigate the case detection rates of both PCR on saliva and nasopharynx and RDT on nasopharynx and saliva, we intend to take four samples, two swabs on saliva, one for RDT and one for PCR, and two swabs on nasopharynx, one for RDT and one for PCR. Patients who have at least one of the common symptoms and who consent to such a procedure will be recruited to compare the four results. Our primary objective is to compare the case detection rates for SARS-CoV-2 of the four testing methods (two sampling types and two test types). Since we may not have RDT and Swissmedic approval at the very start of the trial, we may introduce the different new tests step by step.<br><br>To be precise, we write ‘saliva sample’, but precisely we mean gingivo-buccal swab sample, and this thereafter in the document.

**Objectives:**	**Primary**:<br>To compare the case detection rates for SARS-CoV-2 using two different methods on two different sample types, namely PCR on saliva and nasopharyngeal fluid, and RDT on nasopharyngeal fluid and saliva.<br>**Secondary**:<br>To compare the case detection rate of nasopharyngeal and saliva RDTs, as well as that of saliva PCR stratified by viral load categories measured by nasopharyngeal PCR
**Outcome(s):**	**Primary:**<br>- Proportion of SARS-CoV-2 positive patients for the two different sampling types (saliva vs nasopharyngeal) and two methods (RDT vs PCR) .<br>**Secondary:**<br>- Viral loads of SARS-CoV-2 by PCR on saliva and nasopharyngeal swabs
**Study design:**	Observational prospective comparative trial. Consecutive patients will be enrolled according to inclusion and exclusion criteria.
**Inclusion / Exclusion criteria:**	**Inclusion criteria for enrolment**<br>- Outpatient aged above 18 years who reports having at least one of the following symptoms: reported cough, reported fever, sore throat, reported anosmia, or reported ageusia<br>- Outpatient aged above 18 years who had a close contact with a documented COVID-19 case and presenting with at least one major or one minor symptom (rhinitis, myalgia, headache, fatigue, nausea, vomiting, diarrhoea, abdominal pain, urticaria, vesicules)<br>These criteria correspond to the testing recommendations included in www.coronacheck.ch
	**Exclusion criteria**<br>- Unwilling or incapable of informed consent<br>- Already diagnosed with SARS-CoV-2 in the past<br>- Hospitalized patients<br>- Anticoagulation

**Procedures:**	Patients fulfilling inclusion and exclusion criteria will be recruited consecutively from the two testing sites of Unisanté (Bugnon 44 et Flon) and two testing site in town (Centre medical de la Source and Vidy-Med). After confirmation of inclusion and exclusion criteria (except for antigoagulation that will be asked later), patients will be asked whether they would be willing to provide two saliva samples and one nasopharyngeal swab in addition to that provided for routine testing. After written informed consent, patients will be asked to perform swabbing of the gingiva-buccal fold two times under professional supervision, once for sending to the laboratory to perform PCR, and once for performing the RDT onsite according to the below procedure. They will be taken two nasopharyngeal swabs, one for RDT and one for PCR.<br>The RDTs will be performed and results read according to the manufacturer information (see below).<br>The saliva and nasopharyngeal samples will be handled according to the procedures usually used for nasopharyngeal swabs. Samples will be forwarded to the Institute of Microbiology where they will be analysed by PCR according to the standard procedure (Corman et al 2020, Greub et al 2016, Pillonel 2020).<br>The patient will be considered as positive for SARS-CoV-2 if any of the PCR results or the RDT result on nasopharynx is positive. Indeed, the specificity of these three tests have been shown to be close to 100%. Moreover, in the presence of a suboptimal standard test (PCR on nasopharyngeal swab), it is possible to have higher or differential detection rate with the new tests. The result of the RDT on nasopharynx will be given on the spot because this test has now been validated in a clinical trial (https://www.medrxiv.org/content/10.1101/2020.10.01.20203836v1). The provision of an immediate response may decrease the refusal rate. Since the RDTs are less sensitive than PCR, we will inform patients who receive a negative RDT result that they have to wait for confirmation by PCR analysis before lifting isolation measures. PCR results will be forwarded to the patients using routine channels, the final result being negative if the two PCR and the RDT on nasopharynx are negative, or positive if any of these three tests are positive.<br>Because of problems in RDT availability and need for Swissmedic approval for the Oclin C component, we may start step by step:<br>1) PCR on saliva and nasopharyngeal swab<br>2) When RDT are available: PCR on saliva and nasopharyngeal swab + RDT on nasopharyngeal swab.<br>3) When RDT available and Swissmedic approval granted, all four components, namely PCR on saliva and nasopharyngeal swabs, RDT on saliva and nasopharyngeal swab.

**Intervention:**	In spite of the difficulty to purchase antigen RDT, we were able to receive tests from three different brands that are all CE approved. In order to test the three different brands, and therefore have stronger generalization ability, we will rotate RDT brand use after 33 patients recruited. We started with the RDT from Roche (Standard Q COVID-19) (https://www.roche.com/media/releases/med-cor-2020-09-01b.htm), and then switch to RDTs from Abbott (Panbio COVID-19 Ag) (https://www.globalpointofcare.abbott/en/product-details/panbio-covid-19-ag-antigen-test.html) and then to those from AAZ-LMB (COVID-VIRO) (http://www.covid19aaz.com/). After 99 patients, we will start the cycle again and so on.<br><br>Saliva sample collections will be performed following the guidance published in Ott et al (2020) and Kojima et al (2020). Instead of collecting only saliva in a tube, the patient will perform him//herself a swab of the gingiva-buccal fold, under the supervision of a healthcare worker. A pilot test is ongoing at Unisanté to ensure appropriateness of swab type and procedure. The results of the first 10 positive patients done as a pilot show perfect concordance between PCR results on saliva and nasopharyngeal samples, with 1-3 log viral load less in saliva. Now that we got the RDT, we will follow the pilot on a few samples for RDT analysis on saliva.<br>No coughing or sniffing prior to sample collection is required. Ideally, water should be avoided 10 minutes prior to collection. Other drinks, food, and nasal sprays should be avoided 20 minutes before sample collection.<br><br>The health care worker must wear a personal protection equipment (at minimum, mask and gloves) prior to contact with the patient. The patient must clean his/her hands using alcohol-based sanitizer or soap and water (no fragrances).<br><br>The health care worker should verify all collection material labelling (at minimum, patient name/identifier and date and time of collection) with the patient. While preparing collection materials, he/she directs the patient to begin pooling saliva in his/her mouth.<br><br>If the RDT are available and Swissmedic granted, the following sampling procedure will be performed:<br>1) A first swab will be done on saliva by the patient using the 4 zones (gingiva-buccal fold left and right, tongue and palate) for 30 seconds to perform PCR (see video from1:14 https://coronavirus.delaware.gov/drive-thru-testing-instructions-for-curative-test-english/).<br>2) The second swab will be on saliva collection. The health care worker will open the RDT kit, give the swab to the patient that will pass it for 30 seconds in the 4 zones of the gingiva-buccal fold. The health care worker will perform immediately the RDTs according to the manufacturer instruction as above, e.g (Standard Q COVID-19) (Roche/SD Biosensor : https://www.youtube.com/watch?v=mBdaOHJWxI4).<br>3) The third swab will be done on nasopharyngeal fluid by the health worker to perform PCR (reference test).<br>4) The fourth swab will be done on nasopharyngeal fluid by the health worker to perform the RDT. He will perform immediately the RDTs according to the manufacturer instruction.

	Should the patient refuse the second nasopharyngeal swab, the three other swabs will still be analysed and used for evaluation of detection rate.<br>Following collection, the patient should again clean his/her hands using alcohol-based sanitizer or soap and water. The health care worker will register the samples collection, and place the samples in a secondary container or a biohazard bag with a biohazard label.<br>The health worker will organize the transfer samples at room temperature to the laboratory for sample processing. The virus RNA in saliva remains stable at room temperature for 3-5 days.<br>The health care worker will wait for 15-30 min., read the result, take a picture of the RDT cassette with the tablet, and then transcribe the result in the Redcap database with the tablet. He/she will then inform the patients that has been waiting for the result in a dedicated waiting room.<br>If the RDT are not available, the following sampling procedure will be performed:<br>1) A first swab will be done on saliva by the patient using the 4 zones (gingiva-buccal fold left and right, tongue and palate) for 30 seconds to perform PCR (see video from1:14 https://coronavirus.delaware.gov/drive-thru-testing-instructions-for-curative-test-english/).<br>2) The second swab will be on nasopharyngeal fluid by the health worker to perform PCR (reference test).<br>If the RDT are available, but Swissmedic submission not yet approved, the following sampling procedure will be performed:<br>1) A first swab will be done on saliva by the patient using the 4 zones (gingiva-buccal fold left and right, tongue and palate) for 30 seconds to perform PCR (see video from1:14 https://coronavirus.delaware.gov/drive-thru-testing-instructions-for-curative-test-english/).<br>2) The second swab will be done on nasopharyngeal fluid by the health worker to perform PCR (reference test).<br>3) The third swab will be done on nasopharyngeal fluid by the health worker to perform the RDT. He will perform immediately the RDTs according to the manufacturer instruction.<br>Should the swab included in the RDT kit proves to be inappropriate for saliva collection (too small and therefore insufficient quantity of saliva, which leads to very low detection rate), a thicker swab will be tested. This will be piloted on few known positive samples by nasopharyngeal PCR, as we did in the pilot study for saliva collection for PCR analysis.
**Comparative Intervention :**	Nasopharyngeal swab samples PCR will be collected according to standard procedure (https://www.nejm.org/doi/full/10.1056/nejmvcm2010260). The analysis for PCR will follow standard procedures (Corman et al 2020, Greub et al 2016, Pillonel et al 2020)

**Number of Participants with Rationale:**	**250 SARS-CoV-2 positive patients by any method (RDT on saliva, PCR on saliva, PCR on nasopharynx)**, which corresponds to ~**1250 patients enrolled with**<br>- 1250 saliva samples for RDT<br>- 1250 saliva samples for PCR<br>- 1250 nasopharyngeal samples for RDT<br>- 1250 nasopharyngeal samples for PCR<br>The expected prevalence will be 20% SARS-CoV-2 positive patients by any means (RDT on saliva or nasopharynx, PCR on saliva or nasopharynx)<br>It should be possible to recruit the study subjects relatively easily considering the number of patients who come for testing at Unisanté sites.
**Study Duration:**	12 weeks
**Study Schedule:**	Start of recruitment: 20.09.2020<br>End of recruitment: 24.12.2020
**Investigator(s):**	Overall Coordination<br>- Principle investigator: Prof. Valérie D'Acremont MD PhD<br>- Co-investigators: Profs Blaise Genton MD PhD &. Gilbert Greub MD PhD (laboratory)<br>Leads at Unisanté sites<br>- Site coordinator Bugnon 44: Dr Jean-Marc Schwob<br>- Site coordinator Bugnon Flon: Prof. Nicolas Senn MD PhD<br>- Site coordinator Centre medical de la Source: Dr Gianni Minghelli<br>- Site coordinator Groupe Vidy-Med: Dr Alain Maillard
**Study Centres:**	- Diagnostic testing site at Bugnon 44<br>- Diagnostic testing site at Flon<br>- Diagnostic testing at Centre Médical de la Source<br>- Diagnostic testing at Vidy-Med
**Statistical Considerations:**	An average of 100 patients are tested daily in each Unisanté site. At Unisanté, in week 35, we had 398 tests performed at the Bugnon 44 site with 55 positive (14%), and 749 performed at the Flon site with 80 positives (11%), irrespective of symptoms. At the Centre Médical de la Source and Vidy-Med, at least 100 patients are tested daily too. Given the inclusion criteria, an approximate 20% of the patients should be positive for SARS-CoV-2 (detection rate).<br>The proposed sample size will allow to have a precision of +/-2% on the detection rate based on a confidence interval of 95%.
**GCP Statement:**	This study will be conducted in compliance with the protocol, the current version of the Declaration of Helsinki, the ICH-GCP (as far as applicable) as well as all national legal and regulatory requirements.

STUDY ADMINISTRATIVE STRUCTURE

1.1 Sponsor

**Name**	Unisanté, Centre de médecine générale et santé publique
Address	Unisanté, Rue du Bugnon 44, 1011 Lausanne, Suisse
Telephone	021 314 60 60
Email	Jacques.cornuz@unisante.ch
Role	- Ensuring proper monitoring of the clinical study<br>- Ensuring all the necessary ethic review(s) and approval(s) are obtained<br>- Preparing and submitting clinical trial application(s) and amendment(s) to the appropriate regulatory agencies<br>- Ensuring that any reviewing ethics board and regulatory agencies are promptly informed of any significant new information<br>- Ensuring compliance with labelling, reporting and record-keeping requirements<br>- Ensuring Good Clinical Practice (GCP)

1.2 Principal Investigator and Co-principal investigator

- Overall management
- Interpretation of data, writing of the report
- Securing funding for the clinical trial
- Generating the appropriate clinical trial documentation (for example, informed consent, protocols) and submissions (for example, ethics and/or regulatory submissions)
- Ensuring adequate resources are available for the duration of the trial (for example, experienced staff, investigational and control products, clinical and medical supplies, an analytical laboratory)
- Creating appropriate written procedures (e.g. GCP protocols)
- Meeting all applicable regulatory requirements (e.g. obtaining and maintaining necessary approvals from the relevant ethics review boards)

**Name**	Prof Valérie D'Acremont
Address	**Unisanté**, Département Formation, recherche et innovation<br>Policlinique de médecine tropicale, voyages et vaccinations<br>Rue du Bugnon 44, 1011 Lausanne, Suisse
Telephone	T : +41 21 314 49 32 secr. M :+41 79 556 25 51
Email	Valerie.dacremont@unisante.ch
Role	Principal investigator

**Name**	Prof. Blaise Genton
Address	**Unisanté**, Département Formation, recherche et innovation<br>Policlinique de médecine tropicale, voyages et vaccinations<br>Rue du Bugnon 44, 1011 Lausanne, Suisse

Telephone	+41 79 556 58 68
Email	Blaise.genton@unisante.ch
Role	Co-PI, assist the PI in all study tasks

1.3 Co-Investigator

**Name**	Prof. Gilbert Greub
Address	Laboratoires de microbiologie
Telephone	+41 79 556 17 95
Email	Gilbert.greub@chuv.ch
Role	Laboratory supervisor

1.4 Site coordinators

**Name**	Prof Nicolas Senn
Address	Unisanté, Department of family medicine<br>Rue du Bugnon 44, 1011 Lausanne, Suisse
Telephone	+41 79 556 07 48
Email	Nicolas.senn@unisante.ch
Role	Responsible for the coordination at Flon (clinical aspects)

**Name**	Dr Jean-Marc Schwob
Address	**Unisanté**, Policlinique de médecine tropicale, voyages et vaccinations<br>Rue du Bugnon 44, 1011 Lausanne, Suisse
Telephone	+41 79 556 87 95
Email	Jean-marc.schwob@unisante.ch
Role	Responsible for the coordination at Bugnon 44 (clinical aspects)

**Name**	Dr Alain Maillard
Address	Vidy-Med<br>Route de Chavanne11, 1007 Lausanne, Suisse
Telephone	+41 79 865 81 05
Email	Alain.maillard@vidymed.ch
Role	Responsible for the coordination at Vidy-Med (clinical aspects)

**Name**	Dr Gianni Minghelli
Address	Centre Médical de la Source<br>Avenue Vinet 32, 1004 Lausanne, Suisse

Telephone	+41 21 641 25 32
Email	gianni.minghelli@vidymed.ch
Role	Responsible for the coordination at Centre medical de la Source (clinical aspects)

1.5 Laboratory

**Name**	Dre Katia Jaton PhD PD-MER
Address	Laboratoires de microbiologie
Telephone	+41 79 556 16 80
Email	Katia.jaton-ogay@chuv.ch
Role	Microbiology analyser

1.6 Statistician

**Name**	Mohamed Faouzi
Address	Route de Berne 113
Telephone	+41 21 314 72 87
Email	Mohamed.Faouzi@unisante.ch
Role	Analysis, Interpretation of data

2. ETHICAL AND REGULATORY ASPECTS

Before the study will be conducted, the protocol, the proposed patient information and consent testimony as well as other study-specific documents shall be submitted to the CER-VD, the properly constituted Competent Ethics Committee (CEC) in agreement with local legal requirements, for formal approval. Any amendment to the protocol will be approved (if legally required) by these institutions.

The decision of the CER-VD concerning the conduct of the study will be made in writing to the Principal investigator before commencement of this study. The clinical study for the PCR on saliva and nasopharyngeal as well as the RDT on nasopharyngeal can begin once approval from the CER-VD has been received.

Swissmedic approval will be asked for the conducting the RDT on the saliva 'arm'. The latter will only start after Swissmedic approval. Any additional requirements imposed by the authorities shall be implemented.

2.1 Study registration

This study will be registered in the Clinicaltrials.gov (a registry listed in the WHO International Clinical Trials Registry Platform (ICTRP)).

In addition, registration French will be entered in the Swiss National Clinical trial Portal (SNCTP via BASEC).

2.2 Categorisation of study

OClin category A

- The interventions assessed are the PCR on saliva and the RDT on nasopharyngeal swab against the reference test, which is the PCR on nasopharyngeal swab. RDTs are CE approved.

OClin category C

- The intervention assessed is the RDT on saliva against the reference test, which is the PCR on nasopharyngeal swab. RDT are CE approved.

2.3 Competent Ethics Committee (CEC) and Competent Authorities (CA)

Protocol approval. The principal investigator ensures that approval from an appropriately constituted Competent Ethics Committee (CEC), and Swissmedic is sought for the clinical study OClinC.

Protocol changes. No changes are made to the protocol without prior approval from the Sponsor and CEC, except where necessary to eliminate apparent immediate hazards to study participants. Such urgent changes to the protocol due to immediate hazards must be reported to the Sponsor within 48h. According to OClin art. 37 safety and protection measures which must be taken immediately will be notified to CER-VD.

Unanticipated risks/benefits. This is an observational prospective trial that features very little additional risk to the patient. The collection of saliva swabs is innocuous. The standard procedure for nasopharyngeal swab can be accompanied (very rarely) by lesion of the nasal mucosa. It definitely leads to some discomfort, or even pain, for the patient.

Should this study show a case detection rate for SARS-CoV-2 using RDT on saliva of at least 70% of that using the standard method (naso-pharyngeal PCR), this new method could be proposed as standard diagnostic testing procedure for testing of ambulatory COVID-19 suspected cases to the cantonal and national health authorities, as well as to the national COVID-19 task force. It should ease the work of the testing teams and lead to less

discomfort for patients, and hence probably higher rate of testing readiness from symptomatic patients. The overall testing expenses per person will also be considerably reduced.

The threshold of 70% has been chosen to take into account that RDT on saliva or PCR on saliva are expected to have an overall detection rate lower than that of naso-pharyngeal PCR, but possibly much higher if patients with very low viral loads (e.g 1000 copies/ml) are excluded from the analysis (Jacot et al, in press). The detection of the virus in the latter patients is indeed fluctuating strongly from one sample or one moment to another. Moreover, they probably contribute much less to transmission than patients with higher viral loads.

A particular effort will be made to expedite reporting and wide communication, given the global urgency of the COVID-19 testing situation.

2.4 Ethical Conduct of the Study

The study will be carried out in accordance to the protocol and with principles enunciated in the current version of the Declaration of Helsinki, the guidelines of Good Clinical Practice (GCP) issued by ICH, the Swiss Law and Swiss regulatory authority's requirements. The CEC and regulatory authorities will receive the final report and be informed about study stop/end in agreement with local requirements.

2.5 Declaration of interest

The investigators in this trial have no conflict of interest to declare.

2.6 Patient Information and Informed Consent

Recruitment

- Patients meeting inclusion criteria will be informed about the objectives of the study and given an information sheet. They will be asked for their consent to have a gingivo-buccal and a nasopharyngeal swabbing for PCR, a nasopharyngeal swab for RDT and a gingivo-buccal one for RDT (possibly only two or three swabs, depending on RDT availability and Swissmedic approval). They will be informed about the fact that any positive test (be it from nasopharyngeal PCR, saliva PCR, or nasopharyngeal RDT) will be considered as true positive (and not false positive) because of the excellent specificity of the Ag used for RDT and primers for PCR. This is based also on the principle of precaution since there is no risk of isolating a possible negative symptomatic patient, but there is definitely a public health issue to leave a positive subject being in contact with friends and colleagues.
- No financial compensation will be provided for participation.
- The cost of the routine procedure (nasopharyngeal swab) will be supported by the Direction générale de la santé (DGS) as usual.

2.7 Participant privacy and confidentiality

The investigator affirms and upholds the principle of the participant's right to privacy and that they shall comply with applicable privacy laws. Especially, anonymity of the participants shall be guaranteed when presenting the data at scientific meetings or publishing them in scientific journals.

Individual subject medical information obtained as a result of this study is considered confidential and non-anonymised disclosure to third parties is prohibited.

For data verification purposes, authorised representatives of the Principal investigator, a competent authority (e.g. Swissmedic), or an ethics committee may require direct access to parts of the medical records relevant to the study, including participants' medical history.

2.8 Early termination of the study

The Principal investigator may terminate the study prematurely according to certain circumstances, for example:

- ethical concerns,
- when the safety of the participants is doubtful or at risk, respectively,
- alterations in accepted clinical practice that make the continuation of a clinical trial unwise.

2.9 Protocol amendments

Substantial amendments are only implemented after approval of the CER-VD.

Under emergency circumstances, deviations from the protocol to protect the rights, safety and well-being of human subjects may proceed without prior approval of the sponsor and the CER-VD. Such deviations shall be documented and reported to the sponsor. According to OClin art. 37, safety and protection measures which must be taken immediately will be notified to CER-VD within 7 days.

All non-substantial amendments are communicated to the CER-VD.

An adaptive design may be appropriate in this trial since new tests may become available and also new knowledge accumulates very rapidly and may affect our design or procedures.

3. STATISTICAL METHODS

Statistical analysis will be undertaken jointly by the investigators and Dr Mohamed Faouzi

3.1 Hypothesis

This trial seeks to compare SARS-CoV-2 detection rates by RDT on saliva, RDT on nasopharynx, PCR on saliva and PCR on nasopharynx.

The primary hypothesis is that the proportion of patients detected positive by SARS-CoV-2 RDT is not less than 20% of that of a composite PCR test result (saliva + nasopharynx PCR results combined).

3.2 Determination of Sample Size

**Sample size predicted**	**250 SARS-CoV-2 positive patients** (from either RDT or PCR on saliva and nasopharynx) corresponding to ~**1250** patients enrolled<br>- 1250 RDT on saliva samples<br>- 1250 PCR on saliva samples<br>- 1250 RDT on nasopharyngeal samples<br>- 1250 PCR on nasopharyngeal samples

Currently the number of tests performed daily in the canton de Vaud are >1000 at the different triage sites.

At Unisanté, in week 35, we had 398 tests performed at Bugnon 44 with 55 positive (14%), and 749 performed at the Flon with 80 positives (11%), irrespective of symptoms. At Centre Médical de la Source and Vidy-Med, at least 100 patients are tested every day.

The proposed sample size of 250 positive among 1250 cases tested will allow to have a precision of +/-2% on the detection rate if the latter is 20%, based on a confidence interval of 95%.

Since we may start with only PCR swabs and RDT on nasopharyngeal swab, we may need to slightly increase the sample size to meet our objectives of 250 positives with all four types of samples. A calculation will be performed at the time of interim analysis to potentially adapt the sample size. The protocol will be amended at that time and CER-VD approval sought if there is a modification.

3.3 Statistical Analyses

3.3.1 Primary analysis

Primary outcome:

- Proportion of SARS-CoV-2 positive patients by testing method (RDT vs PCR) and sampling type (saliva vs nasopharyngeal).

Study analysis population

All patients recruited will form the study analysis population. All samples will be analysed, except if a patient withdraws his consent after collection and before analysis of his/her sample. If the microbiological test has already been done, we will keep the result in the overall analysis and the patient will still be informed of his/her result due to the fact that it is the public health benefit that prevails over that of the individual.

The proportion of SARS-CoV-2 positive patients (detection rate) and 95% confidence intervals by any method will be presented. Then the detection rate of each method will be compared to that of all other three methods combined (best possible detection rate, true positives).

Secondary outcome:

- Viral loads (number of copies/ml) of SARS-CoV-2 by sample type, namely PCR saliva and PCR nasopharyngeal swabs

Viral loads will be compared between the two sample collection methods using the Kruskall-Wallis rank test. Pearson correlation coefficient will also be used to compare viral load values using the two methods.

Stratified analysis of detection rates will be performed. Detection rates of the two RDT methods will be calculated according to two virus load categories (<36 Ct value, ≥36 Ct value)

3.3.2 Interim analyses

'After enrolment of 200 patients (~40 positive by any test), an interim analysis will be performed.

In the event that the rapid diagnostic test (RDT) on saliva detects less than 50% of the patients detected by PCR (saliva and nasopharyngeal combined), or more than 20% of those detected by PCR, the 'arm' of RDT on saliva will be stopped, or another RDT type will be tested on saliva.

In the event that the rapid diagnostic test (RDT) on nasopharyngeal swab detects less than 50% of the patients detected by PCR (saliva and nasopharyngeal combined), the 'arm' of

RDT on nasopharyngeal swab will be stopped, or another RDT type will be tested on nasopharyngeal swab.

3.3.3 Deviation(s) from the original statistical plan

In this urgent situation we must be flexible and to respond to important emerging questions on new potential interventions.

Modifications may have to be made to ensure the robustness of the findings, seek confounding factors and respond to emerging trends in the data.

4. ADVERSE EVENTS

4.1 Definition and assessment of adverse and other safety related events

4.1.1 Definition and severity of Adverse events (AEs)

An AE in this case is any untoward medical occurrence in a volunteer, which may occur during or after medical procedure performed with an investigational medical device and does not necessarily have a causal relationship with the intervention. An AE can therefore be any unfavourable and unintended sign (including an abnormal laboratory finding), symptom or disease temporally associated with the study intervention, whether or not considered causally related to the study intervention.

The severity of the adverse events will be recorded according to standard definitions.

4.1.2 Adverse event causality

All AE will be evaluated for causality by the investigational team. In addition, Sponsor (through delegated PV CRO) may make a causality assessment of all SAE. Causality will be assessed according to standard criteria (No relation, possible, probable, and definitely related).

4.2 Actions taken in case of adverse events

All clinically relevant AEs will be monitored and handled according to the medical judgement of the clinical investigator.

The volunteer may be followed up more intensively for the purposes of monitoring the evolution of the AE. For example, a telephonic contact may be transformed into a physical visit so that a clinical exam or further blood samples and investigations relevant for the resolution of the AE may be undertaken. Additionally, the volunteer may be referred for specialist assessment.

All actions will be logged and incorporated into the assessment of AE severity

4.3 Recording and reporting of adverse events

4.3.1 Recording

All AEs (occurring at any point in the trial) that are observed by the Investigator or reported by the volunteer, whether or not attributed to study procedure, will be documented in the eCRF (REDCap) on specific templates developed for this purpose at Unisanté.

4.3.2 Reporting

AEs will be transmitted to the Sponsor. The Principal investigator (or any delegated site staff) will report immediately and within 24 hours all SAE to the sponsor. SAEs will be reported by email via the RedCap survey tool). All elements captured within the SAE form featured in the eCRF will be transmitted. This includes free text and auxiliary information required for

appropriate assessment of the severity and causality of the SAE. A separate email containing relevant coded source documents (laboratory or radiological exams) will be sent simultaneously. SAEs will be reported to the Ethics Committee via BASEC within 7 days by the principal investigator. Swissmedic will also be informed via the sponsor in this time frame.

5. QUALITY ASSURANCE AND CONTROL

5.1 Data handling and record keeping / archiving

5.1.1 Case Report Forms

All protocol-required information will be collected directly from a tablet and entered in REDCAP. Other source documents if necessary will be stored securely. Few variables will be collected, namely ID number, phone number, date of birth, date, sex, duration of symptoms, fever (Y/N), cough (Y/N), anosmia a Y/N), ageusia (Y/N), antigoagulation (Y/N), PCR saliva test result (positive/negative and Viral loads), PCR nasopharyngeal test result (positive/negative and viral loads), RDT nasopharyngeal test result, RDT saliva test result,

Only the Sponsor, PIs, investigators, site coordinators and medical professionals will be allowed to access the source documents and database.

Once identifying information is no longer required for the basic functioning of the trial (i.e. the requirement to identify patient, and telephone for result return), all eCRFs will be strictly anonymised and only the participant number in combination with year of birth will remain.

This identifying information that is removed from eCRFs at the end of participant follow-up will be transferred to a separate secured file which is independent from the eCRFs. And this will be made available only under request to the PI (if the request pertains to pharmacovigilance monitoring, quality assurance reviews, audits or a medical concern of the patient, with the patients consent).

This procedure will be identical for the two new sites, namely the Centre Médical de la Source and Vidy-Med. Indeed, the procedures for result transmission to the patients are handled by the same laboratory for Unisanté and Vidy-Med (Laboratoire de microbiologie). The anonymization and coding can thus be performed in the same manner in all sites.

Only the Sponsor, Investigators, the CER-VD and regulatory authorities will have access to the records.

5.1.2 Specification of source documents

Source documents are original documents, data, and records from which the volunteer's CRF data are obtained. For this study, these will include, volunteer consent electronic signature, medical notes (medical history, vital signs, physical examination, adverse event data, concomitant medication and ID number), laboratory records, and correspondence if any.

Source data will be secured in Unisanté, Centre Médical de la Source and Vidy-Med clinical archives with access granted only to medical professionals participating in the trial.

5.1.3 Record keeping / archiving

The Investigators will maintain appropriate medical and research records for this trial (minimum 10 years), in compliance with ICH E6 GCP and regulatory and institutional requirements for the protection of confidentiality of volunteers. The PI, co-Investigators and medical professionals will have access to records. The Investigators will permit authorized representatives of the Sponsor, as well as ethical and regulatory agencies to examine (and when required by applicable law, to copy) clinical records for the purposes of monitoring, quality assurance reviews, audits and evaluation of the study safety and progress.

5.2 Data management

The Principal Investigator will have the responsibility for overseeing the receiving, entering, cleaning, querying, analysing and storing all data that accrues from the study by designated persons. Data management will be performed by Unisanté. Demographics, clinical data and laboratory results will be integrated into the electronic Data Capture System (Redcap database).

For each batch of data, quality control and triggers to computerized logic and/or consistency checks will be systematically applied in order to detect errors or omissions. After integration of all corrections in the complete set of data, the database will be locked and saved before being released for statistical analysis. Each step of this process will be monitored through the implementation of individual passwords and/or regular backups in order to maintain appropriate database access and to guarantee database integrity.

5.2.1 Data Management System

We will be using a tablet to record the patient ID, name, surname, age, symptoms (fever Y/N, cough Y/N, anosmia Y/N, ageusia Y/N, anticoagulation Y/N, RDT test results (positive/negative), PCR test results (positive/negative) and viral loads. Data will be directly entered into REDCAP database.

5.2.2 Data security, access and back-up

All data collected during this research will be hosted on a secure encrypted server located at Unisanté, Switzerland, for the duration of the proposed research

5.3 Monitoring audits and inspections

The monitoring of the trial will be conducted by the 'Secteur de soutien à la recherche' from the Department of Training, research and innovation at Unisanté.

The study documentation and the source data/documents will be accessible to potential auditors/inspectors (also CER-VD) and questions will be answered during potential inspections. All involved parties must keep the participant data strictly confidential.

5.4 Confidentiality, Data Protection

Once the follow up period of a patient is terminated, namely after patient information of the SARS-CoV-2 PCR test result, all data will be coded. Volunteer data will be identified by a unique study number in the database. A separate independent confidential file containing identifiable information will be stored in a secured location in accordance with data protection requirements. Only the Sponsor representatives, Investigators, the CER-VD and regulatory authorities will have access to the records.

If a study subject is volunteering to communicate with the media, he/she is free to do so on his/her own initiative and responsibility.

The study protocol, documentation, data and all other information generated will be held in strict confidence. No information concerning the study or the data will be released to any unauthorized third party, without prior written approval of the Sponsor. All this information will be given without any names or confidential personal information.

5.5 Storage of biological material and related health data

Biological samples will not be stored after SARS-CoV-2 analysis. According to OClin art. 45, the sponsor will keep all data relating to the clinical trial for at least ten years from the end or the stopping of the clinical trial.

6. PUBLICATION AND DISSEMINATION POLICY

The Investigators will be involved in writing and/or reviewing drafts of the manuscripts, abstracts, press releases and any other publications arising from the study. Given the urgency of the COVID-19 pandemic, every effort will be made to ensure that this is done rapidly. Apart from obvious flaws to the conduct of the study, which may preclude data publication, data will be published under the supervision and authorization of PI and sponsor.

Authorship follows the ICJME principles.

The full anonymised dataset will be made available to scientists on request. The complete statistical code will be published publicly on a GitHub repository. The protocol will be made public as soon as possible to ensure that other groups may create interoperable trials. Until then, it will be shared with all interested scientists privately.

7. FUNDING AND SUPPORT

7.1 Funding

The 'Direction générale de la Santé du canton de Vaud' (DGS) has agreed to pay for the additional PCR tests on saliva (CH 52 francs per sample) and for the RDT (7-30 CHF per test), and this for all sites. The rest of the expenses are those usually charged to DGS for patients who fulfil testing criteria (169 CHF). The extra charges (dedicated staff at Unisanté, data management and analysis) will be taken by research funds at Unisanté.

8. INSURANCE

No insurance is needed for OClinA component because of the absence of risk. In the event of any damage caused to participants in the context of the study, Unisanté will respond to them in its capacity as promoter in accordance with the applicable legal provisions.

9. REFERENCES

Altman DG, Machin D, Bryant TN, Gardner MJ (Eds) (2000) Statistics with confidence, 2nd ed. BMJ Books. (p. 49)

Corman VM, Landt O, Kaiser M, et al. Detection of 2019 novel coronavirus (2019-nCoV) by real-time RT-PCR. Euro Surveill. 2020 Jan;25(3):2000045.

Greub G, Sahli R, Brouillet R, Jaton K. Ten years of R&D and full automation in molecular diagnosis..Future Microbiol. 2016;11(3):403-25.

https://www.nejm.org/doi/full/10.1056/nejmvcm2010260

Jacot D, Greub G, Jaton K, Opota O. Viral load of SARS-CoV-2 across patients and compared to other respiratory viruses. Microbes and Infection, in press

Kojima N, Turner F, Slepnev V, et al. Self-collected oral fluid and nasal swabs demonstrate comparable sensitivity to clinician collected nasopharyngeal swabs for Covid-19 detection. April 15, 2020 (https://www .medrxiv .org/content/10.1101/2020.04.11.20062372v1). preprint.

Ott I, Vogels C, Grubaugh N, Wyllie A (07/01/2020). Saliva Collection and RNA Extraction for SARS-CoV-2 Detection. https://dx.doi.org/10.17504/protocols.io.bh6mj9c6

Pasomsub E, Watcharananan SP, Boonyawat K, et al. Saliva sample as a non-invasive specimen for the diagnosis of coronavirus disease 2019: a cross-sectional study. Clin Microbiol Infect 2020 May 15 (Epub ahead of print).

Pillonel T, Scherz V, Jaton K et al Letter to the editor: SARS-CoV-2 detection by real-time RT-PCR.

Euro Surveill. 2020 May;25(21):2000880.

Vogels CBF, Brackney D, Wang J, et al. SalivaDirect: simple and sensitive molecular diagnostic test for SARS-CoV-2 surveillance. August 4, 2020 https://www.medrxiv.org/content/10.1101/2020.08.03.20167791v1.preprint.

Williams E, Bond K, Zhang B, Putland M, Williamson DA. Saliva as a non-invasive specimen for detection of SARS-CoV-2. J Clin Microbiol 2020; 58(8): e00776-20.

Wyllie AL, Fournier J, Casanovas-Massana A et al. Saliva or Nasopharyngeal Swab Specimens for Detection of SARS-CoV-2. N Engl J Med Sept 1st 2020.

unisanté
Centre universitaire de médecine générale et santé publique • Lausanne

10. APPENDICES

Appendice 1

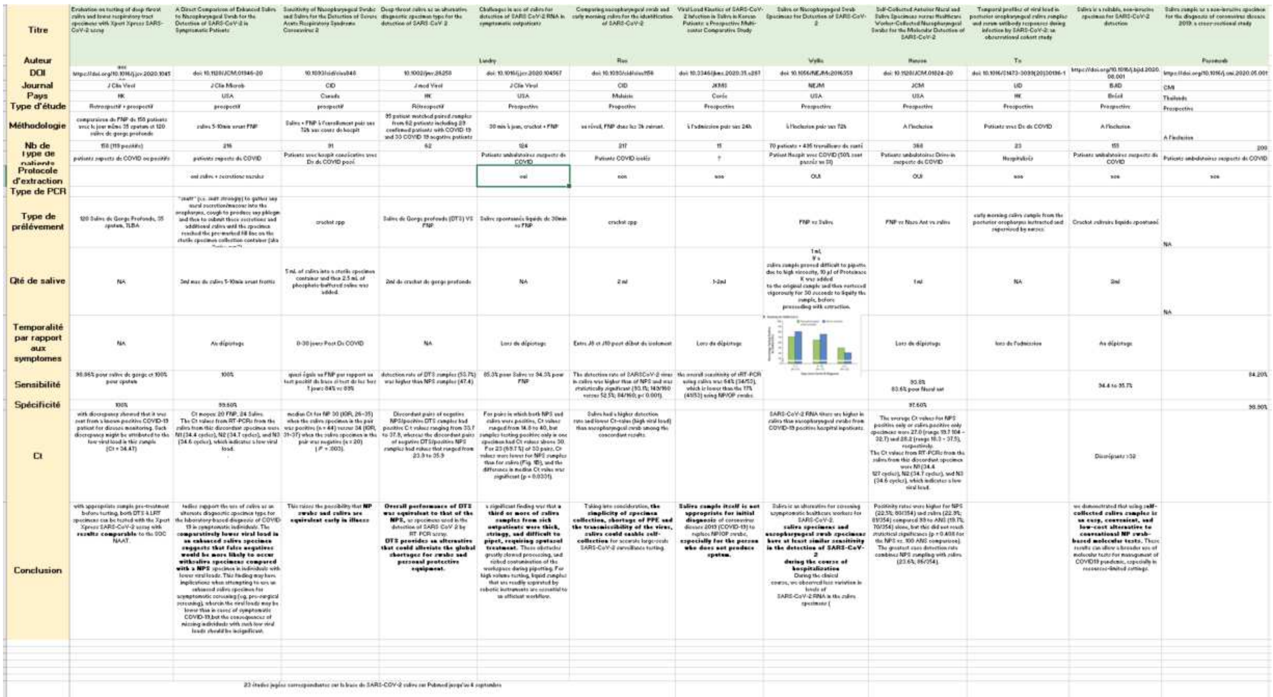

Appendice 2

Colonne1	PANBIO™ COVID-19 Ag	BINAXNOW™ COVID-19 Ag CARD	LumiraDx SARS-CoV-2 Ag Test	Sofia SARS Antigen FIA	STANDARD Q COVID-19 Ag	(2019-nCoV) Antigen Rapid Test Kit
Fabricant	Abbott	Abbott	LumiraDx	Quidel	Sdbiosensor / Roche	Bioeasy
Pays	US	US	UK	US	Corée du Sud / CH	Chine
Certification	CE<br>FDA under an Emergency Use Authorization (EUA)	FDA under an Emergency Use Authorization (EUA)	FDA under an Emergency Use Authorization (EUA)	FDA under an Emergency Use Authorization (EUA)	CE	CE
Mode prélèvement	Frottis NP		Frottis Nasal + NP	Frottis Nasal + NP	Frottis NP	Frottis NP
Mécanisme		Lateral Flow	microfluidic immunofluorescence assay detection of nucleocapsid proteins	immunofluorescence-based lateral flow technology in a sandwich design for qualitative detection of nucleocapsid protein from SARS-CoV-2		Fluorescence Immunochromatographic Assay
Sensibilité	93.3% (98.2% si Ct <33)	97.10%	97.60%	96.70%	96.52%	93.7% (> 98% si Ct<30)
Spécificité	99.40%	98.50%	96.60%	100%	99.68%	100%
Durée min jusqu'au résultat	15 min	15 min	15 min	15 min	15 min	10 min
Durée de possibilité de lecture du résultat	15 - 20 min		15 min	15 min	15 - 30 min	10 min
Nécessité lecteur optique (ou autre)	non	non, mais QR code et application pour le téléphone	oui	oui	non	oui
Durée d'analyse dans le lecteur	/		12 min	1 min	/	30 sec
Disponiblité		Reservé US			late September	arrêt de l'activité
Test/boîte	25	40	12, 24 ou 48	25	25	25
Coût par test		5 USD			7 frs	
Remarques			RT-PCR comparable results within 12 days of onset of symptoms			
Référence industrielle	https://www.globalpointofcare.abbott/en/product-details/panbio-covid-19-ag-antigen-test.html	https://www.abbott.com/BinaxNOW-Test-NAVICA-App.html	https://www.lumiradx.com/assets/images/new/pdf/sars-cov-2-ag-test-strip-product-insert-eua.pdf<br>https://www.lumiradx.com/assets/images/new/pdf/sars-cov-2-ag-qc-product-insert-eua.pdf	https://www.quidel.com/sites/default/files/product/documents/EF1438903EN00.pdf	http://sdbiosensor.com/xe/product/7672	http://en.bioeasy.com.tr/2019-novel-coronavirus-2019-ncov-ag-test-kit-fluorescence-immunochromatographic-assay/
Références scientifiques	interne	interne			interne<br>Sn plutôt à 80%	https://www.ncbi.nlm.nih.gov/pmc/articles/PMC7263236/
Contact	Oui M. Grosse à Abott				Oui, M. Pagès Roche Dx	Oui, 1ère approche Sinoptic
